# Quantitative assessment of rheumatoid arthritis treatment response using short-wave infrared hyperspectral imaging soft abundance scoring

**DOI:** 10.1117/1.JBO.31.4.045001

**Published:** 2026-04-28

**Authors:** Hsin-Hua Chen, Hsin-Che Wang, Kuo-Lung Lai, Chiu-Chin Sung, Jun-Peng Chen, Yu-Wen Fu, Shao-Jen Weng, Hsian-Min Chen

**Affiliations:** a Chung Hsing University, Institute of Biomedical Science, Rong Hsing Research Centre for Translational Medicine and Big Data Center, Taichung, Taiwan; b National Chung Hsing University, Department of Post-Baccalaureate Medicine, College of Medicine, Taichung, Taiwan; c Taichung Veterans General Hospital, Department of Digital Medicine, Taichung, Taiwan; d Taichung Veterans General Hospital, Division of Allergy, Immunology and Rheumatology, Department of Internal Medicine, Taichung, Taiwan; e Taichung Veterans General Hospital, Center for Quantitative Imaging in Medicine (CQUIM), Department of Medical Research, Taichung, Taiwan; f National Taiwan University, Graduate Institute of Biomedical Electronics and Bioinformatics, Taipei, Taiwan; g Taichung Veterans General Hospital, Department of Medical Research, Biostatistics Task Force, Taichung, Taiwan; h Tunghai University, Department of Industrial Engineering and Enterprise Information, Taichung, Taiwan; i National Chung Hsing University, Doctoral Program in Translational Medicine, Taichung, Taiwan; j National Chung Hsing University, Rong Hsing Translational Medicine Research Center, Taichung, Taiwan

**Keywords:** rheumatoid arthritis, optical measure, short-wave infrared hyperspectral imaging, hyperspectral imaging soft abundance scorer, inflammation quantification

## Abstract

**Significance:**

Non-invasive optical measures for evaluating rheumatoid arthritis (RA) remain limited. Clinical scores such as the 28-joint Disease Activity Score (DAS28) and ultrasound are operator-dependent and do not directly quantify optical tissue changes. Short-wave infrared (SWIR) hyperspectral imaging (HSI) provides sensitivity to inflammation-associated spectral changes in biological tissues.

**Aim:**

The aim is to develop and evaluate a quantitative HSI soft abundance scoring (HSISAS) method for assessing treatment response in RA patients using SWIR-HSI.

**Approach:**

Eleven RA patients who met the 2010 American College of Rheumatology/European Alliance of Associations for Rheumatology criteria underwent SWIR-HSI (900 to 1700 nm) of the wrist joints before and after 12 weeks of biologic therapy. The HSISAS algorithm used pixel-wise reflectance spectra together with spectral correlation structure estimation and constrained energy minimization-based abundance scoring to compute intra- and inter-subject scores. These scores were statistically compared with conventional clinical indices, including DAS28, erythrocyte sedimentation rate, C-reactive protein, and power Doppler ultrasound findings.

**Results:**

Both intra- and inter-HSISAS significantly increased after treatment (p<0.001) and were positively associated with DAS28 improvement. Reflectance spectra showed visible pre/post-treatment differences, particularly near 1250 nm. The abundance maps visually differentiated pre- and post-treatment states consistent with clinical improvement.

**Conclusions:**

The HSISAS framework provides a quantitative, non-contact optical measure of treatment-related spectral changes in RA. This method may offer translational potential for disease monitoring and may complement existing ultrasound and laboratory assessments.

## 1 Introduction

Rheumatoid arthritis (RA) is a chronic inflammatory joint disease with a prevalence of ∼1% and a female predominance of ∼3:1.^1^^,^^2^ It primarily affects small joints such as those of the hands, wrists, knees, and feet, with the hands being most frequently and severely involved. RA shortens life expectancy by 1 to 10 years compared with the general population,^3^ mainly due to cardiovascular,^4^ pulmonary,^5^ and ocular complications,^6^ underscoring the need for early and accurate disease assessment.

Diagnosis of RA follows the 2010 American College of Rheumatology/European Alliance of Associations for Rheumatology (ACR/EULAR) criteria, which consider the number of affected joints, serologic markers, acute inflammatory indicators, and symptom duration. The 28-Joint Disease Activity Score (DAS28) quantifies the clinical disease activity based on the tender joint count, swollen joint count, patient self-assessment of general health, and inflammatory markers such as erythrocyte sedimentation rate (ESR) or C-reactive protein (CRP).^7^ Although DAS28 and musculoskeletal ultrasonography are routinely used, both depend on operator expertise and subjective scoring, limiting reproducibility and standardization. Therefore, an objective, quantitative, and non-contact method for assessing joint inflammation is desirable.

During the COVID-19 pandemic, there has been an increasing need for objective, non-contact tools to evaluate RA disease activity. Hyperspectral imaging (HSI), an optical remote sensing technology, offers a promising alternative. HSI captures hundreds of continuous spectral bands and combines high spatial and spectral resolution to detect material signatures invisible to the naked eye. HSI has shown potential in biomedical material detection,^8^ food safety,^9^^,^^10^ burn assessment,^11^^–^^13^ and biomedicine,^14^^,^^15^ making it a valuable tool for tissue characterization and optical diagnosis.

Because the effective sampling depth of diffuse reflectance imaging in the short-wave infrared (SWIR, 900 to 1700 nm) range is not fixed, it is influenced by wavelength, tissue composition, and imaging geometry. Previous work by Zhang et al.^16^ showed that wavelengths around 1300 to 1375 nm provide relatively favorable penetration for deeper tissue assessment, and the 1550- to 1600-nm range also demonstrated good penetration performance in highly pigmented tissues such as the liver. In addition, validation studies using different phantoms have shown that SWIR-HSI can detect targets located at millimeter-to-centimeter depths in collagen phantoms^17^ and chicken breast tissue,^18^ supporting the technical penetration capability of this spectral range. Based on these findings, SWIR-HSI may serve as a feasible non-contact optical approach for assessing superficial and subsurface periarticular inflammation-related tissue changes associated with RA.

The SWIR range is also considered a suitable spectral window for biological tissue assessment because it combines relatively reduced tissue scattering with characteristic absorption features from water, lipids, and collagen-related tissue components.^19^^,^^20^ In particular, water exhibits prominent absorption bands near 970, 1190, and 1450 nm, whereas lipids show characteristic features around 1040, 1210, and 1400 nm within the SWIR region.^19^ These optical properties make SWIR sensitive to variations in tissue composition and microstructure. In RA, synovitis and periarticular inflammation are commonly associated with edema, increased vascular permeability, and tissue remodeling, which may alter local optical properties related to water, lipids, and collagen.^21^ Therefore, SWIR hyperspectral imaging may provide a non-invasive optical means to characterize superficial and subsurface periarticular tissue changes in the wrist region that are associated with inflammation.

Prior studies, such as Milanic et al.,^22^ demonstrated the feasibility of HSI in detecting RA by analyzing optical transmission in healthy and arthritic joints using Monte Carlo simulations. They found that spectral ranges of 800 to 900 nm and 1050 to 1100 nm effectively distinguished healthy from diseased joints, particularly for early detection.^22^ Similarly, Putten et al.^23^ explored hypoxia’s role in RA progression using multispectral oximetry, finding significant differences in oxygen saturation between healthy and inflamed tissues. Despite these advances, existing studies primarily rely on simulations, which are computationally intensive and unsuitable for real-time clinical applications.

To overcome these limitations, we developed an intra- and inter-subject hyperspectral imaging soft abundance scoring (HSISAS) method for quantitative assessment of treatment-related spectral changes in RA. The HSISAS algorithm uses pixel-wise reflectance spectra from the wrist regions together with spectral correlation structure estimation and constrained energy minimization (CEM)-based abundance scoring^24^^,^^25^ to quantify treatment-related spectral variability within and across subjects. By comparing HSISAS results with clinical indices such as DAS28 and quantitative ultrasound measurements before and after 12 weeks of biologic therapy, this study aims to evaluate HSISAS as a non-invasive optical measure of treatment-related wrist spectral changes and to examine its association with clinical disease activity in RA patients.

## 2 Materials and Methods

### 2.1 Subject Recruitment and Clinical Data Collection

**Fig. 1:**
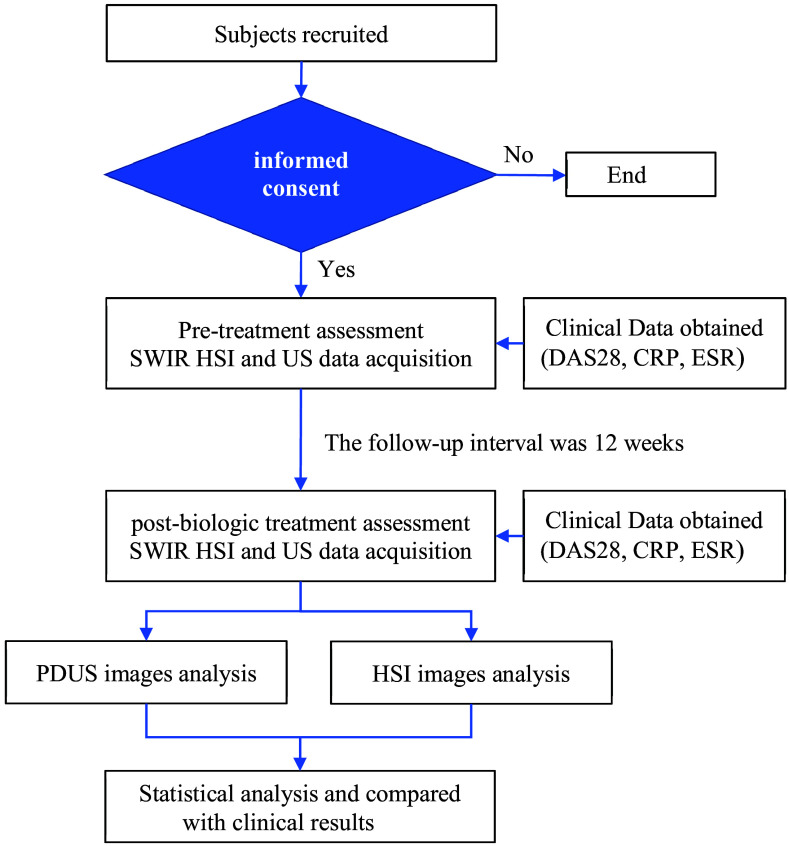
Study analysis flowchart.

This study enrolled 11 RA patients (3 males and 8 females; mean age: 56.5±10.2 years) meeting the ACR/EULAR criteria and receiving biologic therapy. The study was approved by the Institutional Review Board of Taichung Veterans General Hospital (Approval No. CE22138B), and all procedures were conducted according to the relevant guidelines and regulations, including the principles outlined in the Declaration of Helsinki. Written informed consent was obtained from all participants before their inclusion. Clinical data were collected before treatment and after 12 weeks, including DAS28, ESR, CRP, and power Doppler ultrasonography (PDUS) of hand joints. Short-wave infrared hyperspectral imaging (SWIR-HSI) of the wrists was also performed at both time points. The study workflow is summarized in Fig. 1.

### 2.2 Analysis for Ultrasound Imaging of the Joints in Patients with RA

**Fig. 2:**
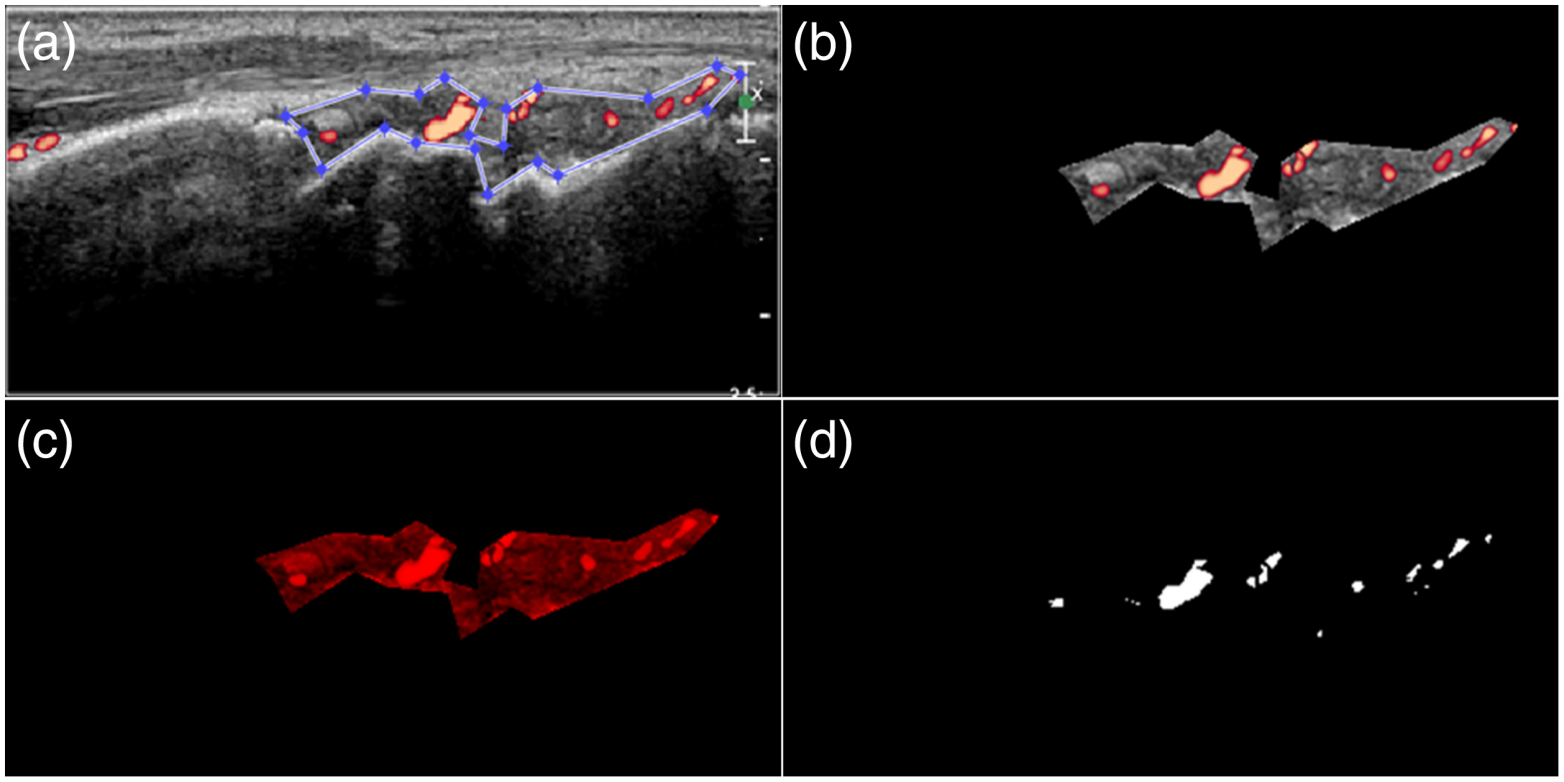
Computer-aided quantitative analysis of PD ultrasound image of the wrist. (a) Original image shows color flows in the hypertrophied synovium. The boundary of the synovium is delineated manually. (b) Segmentation of the synovium. (c) Decomposition of the RGB image and obtaining a red-gray image. (d) Convert the red-gray image to a binary image with an appropriate threshold then calculate the area of PD.

The hand joints, including the bilateral thumb IP, PIP2-5, MCP1-5, and bilateral wrists, were assessed with PDUS at weeks 0 and 12 of biological therapy. An ultrasound system, Philips iU22 (Philips Ultrasound, Bothell, Washington, United States), equipped with a 12-MHz linear probe and adopted appropriate power Doppler (PD) settings: pulse repetition frequency 500 Hz and wall filter 47 Hz, was used for ultrasound assessment. The room temperature was kept at 25°C during the ultrasound assessments. Patients were instructed to rest, including 15 min of joint immobility, to prevent exercise-induced articular hyperemia. The hands were positioned on the table in a standardized manner and scanned longitudinally at the dorsal side of the joints.^26^ The color gain was adjusted until color pixels were no longer observable beneath the bone cortex to optimize the PDUS images. The transducer should not compress the skin to avoid generating false negatives. All PDUS images were saved digitally in PNG file format. Quantitative image analysis was done using the MATLAB software (version R2018b, The MathWorks, Natick, Massachusetts, United States). The region of interest (ROI) of the synovium was determined manually by an experienced rheumatologist and then was segmented for calculating the synovial area, followed by red, green, and blue (RGB) decomposition and conversion of the red-gray image to the binary image for calculating the PD area (Fig. 2).

### 2.3 SWIR Hyperspectral Imaging System

A push-broom hyperspectral imaging system was used, consisting of an ImSpector N17E spectrometer (SPECIM, Oulu, Finland), an OW1.7-VS-CL-640 InGaAs detector (Raptor Photonics, Leuven, Northern Ireland), and a Kowa LM12HC-VIS-SW lens (Kowa, Japan; focal length: 12 mm; maximum aperture: F1.8). The system covered a spectral range of 900 to 1700 nm, with a spatial image size of 640×635  pixels, a spectral resolution of 5 nm using a 30-μm slit, and 512 spectral bands. Illumination was provided by two 50-W halogen lamps (OSRAM DecoStar 51 ALU, Germany).

To reduce acquisition-related variability, all subjects were imaged under a standardized and fixed imaging geometry. The working distance between the lens and the wrist was fixed at 25 cm. During data acquisition, the lens aperture was fixed at F4, and the focus ring was fixed at 0.2 m. The wrist was positioned on an imaging platform covered with a green board marked with 1×1  cm grids, which served as a visual reference for consistent hand placement and white-reference positioning. The two halogen lamps were arranged in a bilateral oblique illumination geometry based on our prior clinical imaging experience and aligned to predefined marks to provide consistent illumination across subjects.

**Fig. 3:**
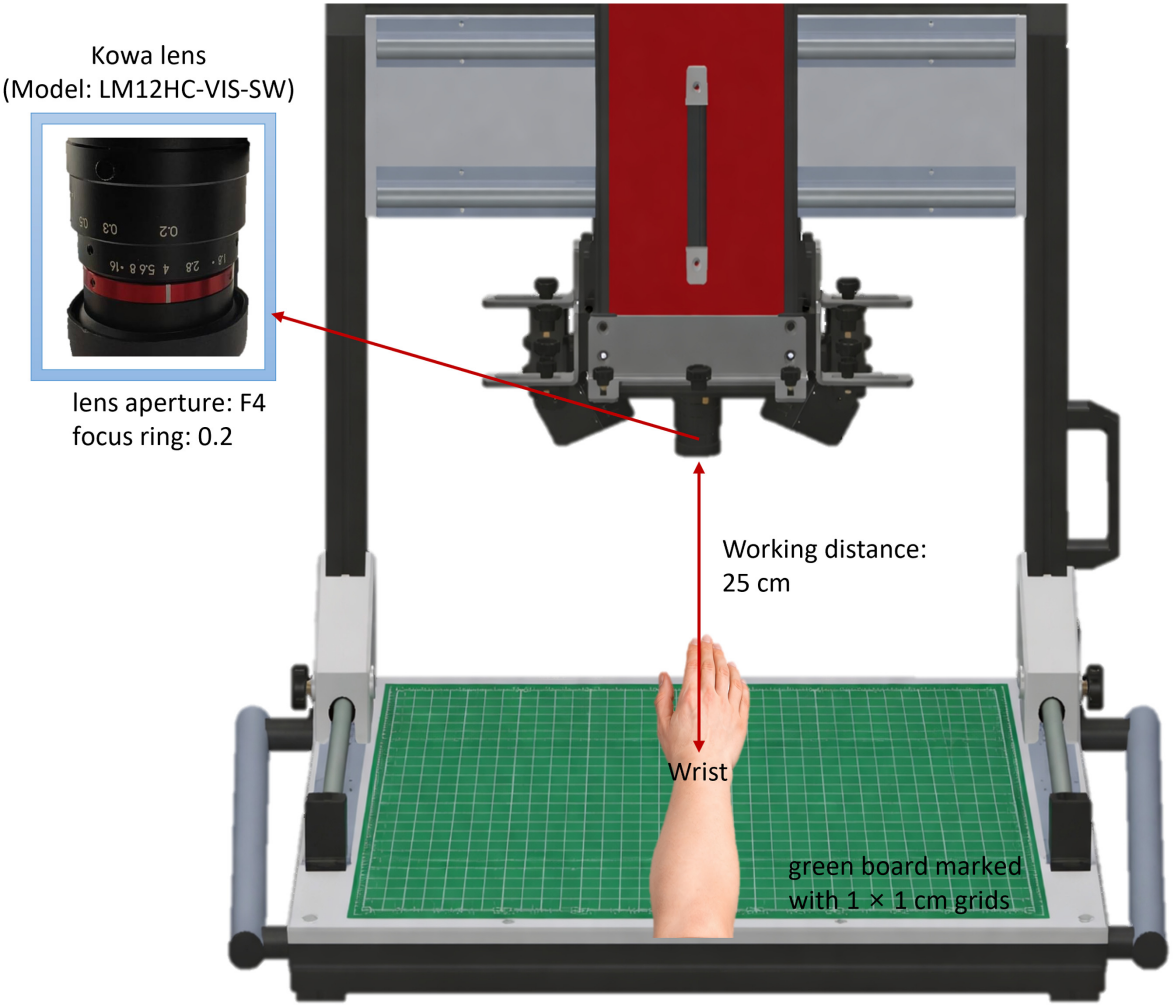
Standardized SWIR hyperspectral imaging setup for RA wrist acquisition, showing the fixed working distance (25 cm), lens setting, wrist placement, and green imaging board marked with 1×1  cm grids used to maintain consistent acquisition geometry.

The exposure (integration) time was fixed at 58 ms, and image acquisition was performed at a frame rate of 15  frames/s. For each subject, a total of 635 frames were collected, corresponding to a scan duration of ∼42.33  s. Before each imaging session, the hyperspectral imaging system and illumination sources were warmed up for ∼20  min to improve measurement stability. For each subject, the wrist hyperspectral image was acquired first, followed by white-reference acquisition using a standard white board placed at the predefined location, and then dark-reference acquisition with the lens cap closed. The calibrated reflectance image for each subject was subsequently calculated from the raw hyperspectral image using the corresponding white and dark references before entering the preprocessing step. These procedures were used to reduce variability related to imaging distance, illumination conditions, and system response prior to subsequent spectral analysis. A schematic illustration of the standardized imaging setup, including the fixed working distance, lens setting, wrist placement, and gridded imaging platform, is shown in Fig. 3.

### 2.4 Short-Wave Infrared Hyperspectral Imaging Analysis Methods

**Fig. 4:**
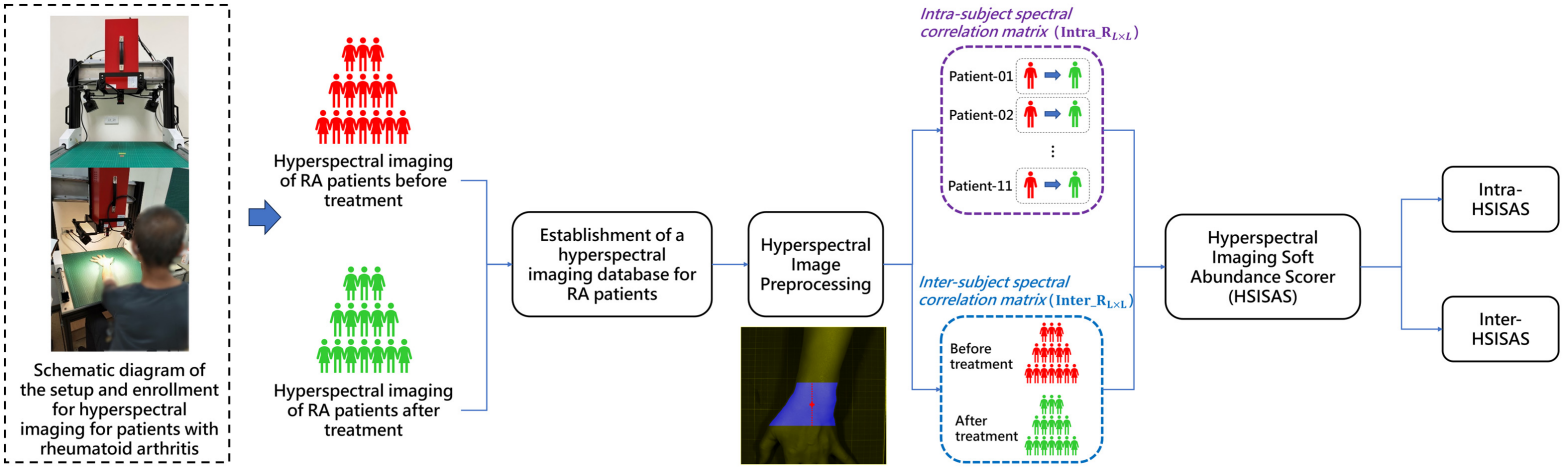
Workflow of the proposed HSISAS framework for RA patients before and after treatment, including preprocessing, spectral correlation matrix construction, and abundance scoring.

Hyperspectral imaging data were analyzed using a modified HSISAS algorithm.^27^ The analysis involved three key steps: (1) preprocessing to extract wrist joint regions, (2) computing correlation matrices for individual and group RA patients before and after treatment, and (3) calculating intra- (Intra-HSISAS) and inter-subject (Inter-HSISAS) abundance scores for statistical analysis with clinical data. The hyperspectral imaging algorithm analysis workflow for RA patients is shown in Fig. 4.

#### 2.4.1 Preprocessing

After acquiring hyperspectral images, preprocessing was conducted to segment the wrist joints. The band ratio method (1303  nm/1453  nm) enhanced contrast between the hand and background. Otsu’s algorithm then binarized the image for segmentation. A rheumatologist manually selected wrist coordinates, and 100 pixels were extended vertically to extract the wrist region for further analysis.

#### 2.4.2 Calculation of spectral correlation matrices for individual and group RA subjects before and after treatment

Following preprocessing, the wrist joint regions were extracted from pre- and post-treatment hyperspectral images. The HSISAS framework was then applied to derive two quantitative indices: Intra-HSISAS, which characterizes within-subject spectral changes before and after treatment, and Inter-HSISAS, which characterizes the similarity of spectral changes at the group level.

In this study, the input data for HSISAS consisted of the pixel-wise reflectance spectra extracted from the segmented wrist regions. Each pixel spectrum was represented as a spectral vector with dimension 1×L, where L is the number of spectral bands. Based on these spectral vectors, individual-level and group-level spectral correlation matrices were calculated using Eqs. (1) and (2), respectively

$$\text{Intra}\_R_{L\times L}=\frac{\sum_{i=1}^{n}(x_{i}-\overset{¯}{x})(y_{i}-\overset{¯}{y})}{\sqrt{\left[\sum_{i=1}^{n}(x_{i}-\overset{¯}{x}{)}^{2}][\sum_{i=1}^{n}(y_{i}-\overset{¯}{y}{)}^{2}\right]}},$$ (1)

$$\text{Inter}\_R_{L\times L}=\frac{\sum_{k=1}^{nm}(x_{k}-\overset{¯}{x})(y_{k}-\overset{¯}{y})}{\sqrt{\left[\sum_{k=1}^{nm}(x_{k}-\overset{¯}{x}{)}^{2}][\sum_{k=1}^{nm}(y_{k}-\overset{¯}{y}{)}^{2}\right]}}.$$ (2)

For the intra-subject analysis, $x_{i}$ and $y_{i}$ denote the spectral vectors of the wrist region from the same RA subject before and after treatment, respectively, and $\overset{¯}{x}$ and $\overset{¯}{y}$ denote the corresponding mean spectral vectors. For the inter-subject analysis, the spectral information from all enrolled RA subjects was combined to estimate the group-level correlation structure. Here, n represents the total number of pixels in the wrist joint regions of both hands for one subject, and m represents the total number of RA subjects. These correlation matrices summarize the spectral relationships used in the subsequent CEM-based abundance scoring.

#### 2.4.3 Improved version of the HSISAS

An improved HSISAS, based on the CEM method,^27^ was used to quantify treatment-related spectral changes in RA wrist tissues. The scoring function is defined as shown in Eq. (3)

$$\delta^{\mathrm{HSISAS}}(r)=\frac{{\mathrm{rR}}_{L\times L}^{-1}d}{d^{T}R_{L\times L}d}.$$ (3)

In this framework, r denotes the set of spectral vectors for all pixels in the hyperspectral image, and d denotes the target spectral signature. In the present study, d was defined as the mean post-treatment spectral vector derived from the wrist joint regions of the left and right hands, representing an internal reference spectral pattern associated with a relatively improved inflammatory state. This target definition was used for within-cohort treatment-related similarity scoring and should not be interpreted as an external normative standard. The matrix $R_{L\times L}$ denotes the spectral correlation matrix used in the CEM framework. In this study, the intra-subject and inter-subject correlation matrices obtained from Eqs. (1) and (2) were substituted into Eq. (3) to generate the intra- and inter-subject HSISAS results, as shown in Eqs. (4) and (5)

$$\text{Intra}\_\mathrm{HSISAS}(r)=\frac{r\mathrm{Intra}\_R_{L\times L}^{-1}d}{d^{T}\text{Intra}\_R_{L\times L}^{-1}d},$$ (4)

$$\text{Inter}\_\mathrm{HSISAS}(r)=\frac{r\text{Inter}\_R_{L\times L}^{-1}d}{d^{T}\text{Inter}\_R_{L\times L}^{-1}d}.$$ (5)

Accordingly, the resulting HSISAS value reflects the degree of spectral similarity between the measured wrist spectrum and the target spectral signature under the correlation structure defined by $R_{L\times L}$. A higher HSISAS value indicates that the measured spectrum is more similar to the post-treatment reference pattern, whereas a lower value indicates greater spectral deviation from that reference. Therefore, in the present study, HSISAS should be interpreted as a quantitative optical similarity measure of inflammation-related tissue changes, rather than as a direct measurement of a single biochemical constituent.

From a physiological perspective, Intra-HSISAS reflects within-subject spectral change and is intended to quantify how closely the wrist spectra of an individual patient approach the post-treatment reference state. Inter-HSISAS reflects group-level spectral similarity and is intended to characterize how closely a patient’s spectral pattern resembles the overall post-treatment behavior observed across subjects. In the context of RA, these scores are interpreted as optical indicators associated with treatment-related changes in tissue hydration, scattering behavior, and microstructural remodeling.

To improve the clarity and reproducibility of the mathematical formulation, the main symbols used in Eqs. (1)–(5) are summarized in Table 1.

**Table 1:** Notation used in the HSISAS formulation.

Symbol	Definition
r	The set of spectral vectors for all pixels in the hyperspectral image
d	Target spectral signature used in the CEM-based HSISAS analysis
$R_{L\times L}$	Spectral correlation matrix used in the CEM framework
$x_{i}$	Spectral vector of the wrist region before treatment
$y_{i}$	Spectral vector of the wrist region after treatment
$\overset{¯}{x}$, $\overset{¯}{y}$	Mean spectral vectors before and after treatment, respectively
L	Number of spectral bands
n	Total number of pixels in the wrist ROIs of both hands for one subject
m	Total number of RA subjects

### 2.5 Statistical Analysis

In this study, the region of interest of the synovium (ROI_Syn) and PD area calculated from ultrasound images, the intra- and inter-subject hyperspectral imaging soft abundance scorer results (Intra_HSISAS and Inter_HSISAS), and clinical data (DAS28, ESR, and CRP) were expressed using the median and interquartile range (IQR). Changes before and after treatment were analyzed using the Wilcoxon signed-rank test and Spearman correlation in SPSS 22.0 (p<0.05).

Hyperspectral imaging and ultrasound measurements were analyzed at the wrist level because the left and right wrists were imaged separately for each patient. Therefore, each patient could contribute up to 2 wrist-level observations at each visit, resulting in 22 wrist-level observations from 11 patients at each time point. By contrast, DAS28 is a patient-level clinical measure rather than a wrist-level variable. For DAS28-related analyses, the DAS28 score obtained at each visit was assigned to the bilateral wrist images acquired on the same day. If DAS28 was not assessed on the imaging day, the corresponding value was treated as missing data and was excluded from DAS28-related paired analyses and correlation analyses.

## 3 Results

### 3.1 Comparison of Clinical Data Before and After Treatment

This study used the Wilcoxon signed-rank statistical test to analyze the ultrasound imaging data, hyperspectral imaging data, and clinical indicator data before and after treatment, as shown in Table 2. Post-treatment, the ROI_Syn area significantly decreased (p=0.020), from 13,904.8 pixels (IQR 5868.4 to 17,277.5) to 8290.2 pixels (IQR 5646.2 to 17,008.9), indicating a significant reduction in the synovial area. Although the PD area decreased from 331.1 to 195.3 pixels after treatment, this change was not statistically significant (p=0.394). Intra_HSISAS (intra-subject abundance score) significantly increased from 0.2 (IQR 0.2 to 0.3) to 0.9 (IQR 0.9 to 0.9) (p<0.001). Inter_HSISAS (inter-subject abundance score) also significantly increased from 0.6 (IQR 0.4 to 0.8) to 0.9 (IQR 0.7 to 1.0) (p<0.001), showing significant improvement in abundance scores derived from hyperspectral imaging data post-treatment. Post-treatment, DAS28 significantly decreased (p=0.003), from 4.8 (IQR 4.2 to 5.5) to 4.3 (IQR 3.3 to 4.8), indicating a significant improvement in the clinical disease activity index. ESR and CRP both significantly decreased, with ESR dropping from 31.0 (IQR 12.0 to 75.0) to 19.0  mm/h (IQR 8.0 to 32.0) (p=0.002) and CRP from 1.5 (IQR 0.2 to 1.8) to 0.2  mg/dL (IQR 0.0 to 1.1) (p<0.001), indicating a significant reduction in inflammatory responses post-treatment.

**Table 2:** Comparison of ultrasound imaging, intra- and inter-subject hyperspectral imaging soft abundance scores, and inflammatory indices before and after treatment in patients with rheumatoid arthritis.

	Pre (n=22)		Post (n=22)		p-value
ROI_Syn area (pixels)	13,904.8	(5868.4, 17,277.5)	8290.2	(5646.2, 17,008.9)	0.020*
PD area (pixels)	331.1	(0.0, 1146.8)	195.3	(0.0, 713.8)	0.394
Intra_HSISAS	0.2	(0.2, 0.3)	0.9	(0.9, 0.9)	<0.001**
Inter_HSISAS	0.6	(0.4, 0.8)	0.9	(0.7, 1.0)	<0.001**
DAS28 (n=16)	4.8	(4.2, 5.5)	4.3	(3.3, 4.8)	0.003**
ESR (mm/h)	31.0	(12.0, 75.0)	19.0	(8.0, 32.0)	0.002**
CRP (mg/dL)	1.5	(0.2, 1.8)	0.2	(0.0, 1.1)	<0.001**

Wilcoxon signed rank: *p<0.05 and **p<0.01.

ROI_Syn, region of interest of synovium; PD, power Doppler; Intra_HSISAS, intra-hyperspectral imaging soft abundance score; Inter_HSISAS, inter-hyperspectral imaging soft abundance score; DAS28, 28-Joint Disease Activity Score; ESR, erythrocyte sedimentation rate; CRP, C-reactive protein

Values are reported at the wrist level. The pre-treatment and post-treatment columns each include 22 wrist-level observations from 11 patients. DAS28 is a patient-level variable; only cases with both pre- and post-treatment DAS28 assessments were included in DAS28-related analyses (n=16 wrist-level entries from 8 patients).

### 3.2 Correlation Analysis of Treatment-Related Changes in Ultrasound, HSISAS, and Clinical Data in Patients with RA

To improve clinical interpretability, correlation analysis was performed using improvement-oriented change variables. For variables expected to decrease after treatment, including ROI_Syn, PD, DAS28, ESR, and CRP, change was defined as pre-treatment minus post-treatment values. For variables expected to increase after treatment, including Intra_HSISAS and Inter_HSISAS, change was defined as post-treatment minus pre-treatment values. Spearman’s rho correlation analysis was then conducted, and the results are shown in Table 3.

**Table 3:** Spearman’s rho correlation coefficients for improvement-oriented changes in ultrasound imaging, HSISAS scores, and inflammatory indices. Ultrasound and HSISAS variables were analyzed at the wrist level. DAS28 is a patient-level variable, and cases with missing pre- or post-treatment DAS28 values were excluded from DAS28-related analyses.

	ΔROI_Syn	ΔPD	ΔIntra_HSISAS	ΔInter_HSISAS	ΔDAS28	ΔESR	ΔCRP
	$r_{s}$	$r_{s}$	$r_{s}$	$r_{s}$	$r_{s}$	$r_{s}$	$r_{s}$
ΔROI_Syn	—						
ΔPD	−0.069	—					
ΔIntra_HSISAS	−0.488*	0.298	—				
ΔInter_HSISAS	−0.315	0.444*	0.669**	—			
ΔDAS28	−0.154	0.059	0.669**	0.746**	—		
ΔESR	−0.195	0.120	0.125	0.387	0.381	—	
ΔCRP	−0.387	0.007	0.238	0.256	0.333	0.755**	—

Spearman’s rho coefficient: *p<0.05 and **p<0.01.

Using this sign convention, both Intra_HSISAS improvement and Inter_HSISAS improvement showed significant positive correlations with DAS28 improvement (r_s = 0.669 and 0.746, respectively; p<0.01), indicating that greater hyperspectral improvement was associated with greater improvement in clinical disease activity. Inter_HSISAS improvement was also significantly positively correlated with PD improvement (r_s = 0.444, p<0.05), suggesting an association between improvement in inter-subject hyperspectral spectral similarity and reduction in power Doppler signals.

In addition, Intra_HSISAS improvement and Inter_HSISAS improvement were highly significantly positively correlated with each other (r_s = 0.669, p<0.01), indicating that the treatment-related spectral changes quantified by the intra- and inter-subject HSISAS measures were broadly consistent. By contrast, ROI_Syn improvement showed a significant negative correlation with Intra_HSISAS improvement (r_s = −0.488, p<0.05). This finding should be interpreted cautiously, as it may suggest that ultrasound-based synovial area reduction and HSISAS-based spectral improvement capture partially overlapping but not identical aspects of treatment response in this pilot cohort.

No significant correlations were observed between HSISAS improvement and ESR or CRP improvement. However, ESR improvement and CRP improvement remained significantly positively correlated with each other (r_s = 0.755, p<0.01), which is consistent with the expected relationship among systemic inflammatory markers.

**Fig. 5:**
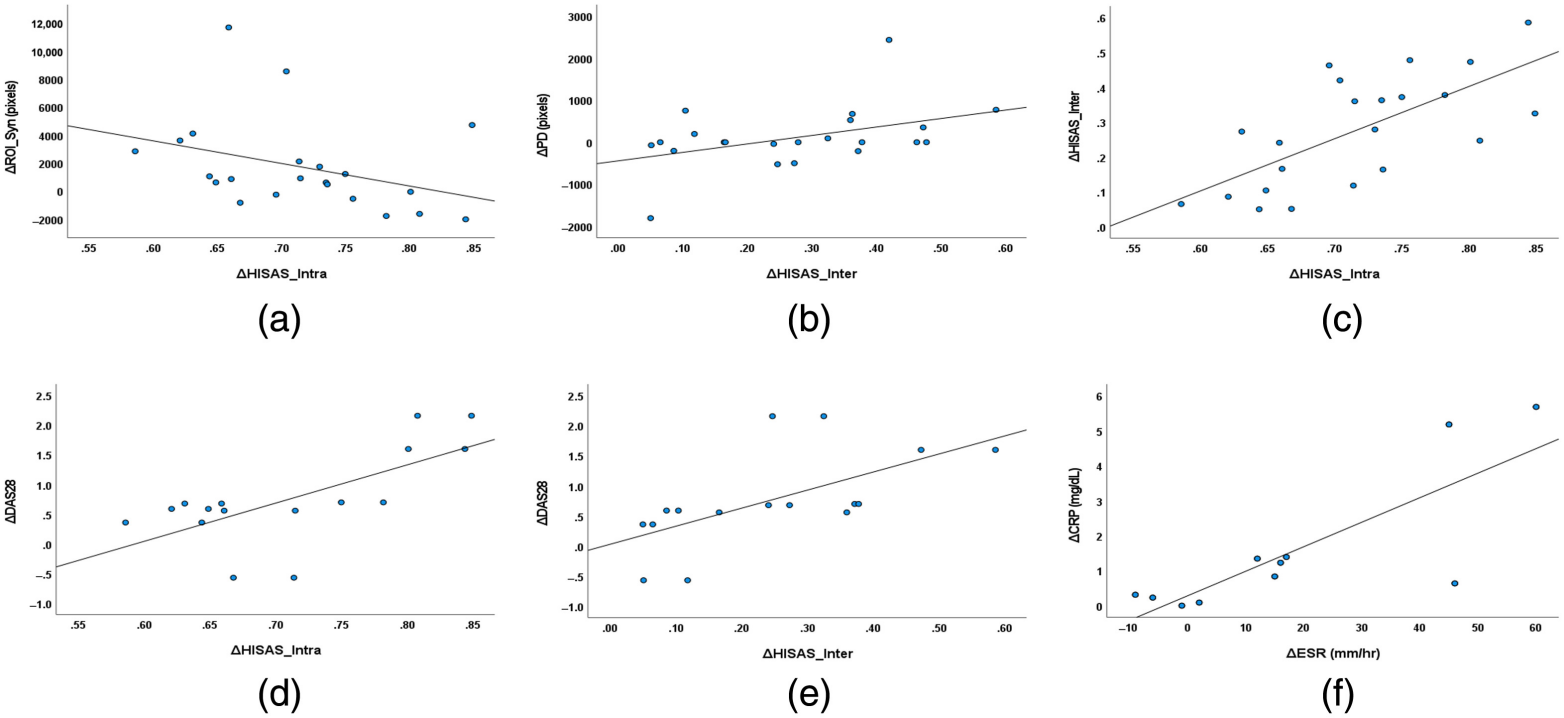
Scatterplots of the statistically significant treatment-related associations identified in Table 3, including (a) ΔIntra_HSISAS versus ΔROI_Syn, (b) ΔInter_HSISAS versus ΔPD, (c) ΔIntra_HSISAS versus ΔInter_HSISAS, (d) ΔIntra_HSISAS versus ΔDAS28, (e) ΔInter_HSISAS versus ΔDAS28, and (f) ΔESR versus ΔCRP. All plots were generated using the same improvement-oriented change variables as those used in the Spearman correlation analysis.

To facilitate visual assessment of the reported treatment-related associations, scatterplots corresponding to the statistically significant correlations in Table 3 are shown in Fig. 5. These plots were generated using the same improvement-oriented change variables as those used in the Spearman correlation analysis and provide a direct view of the data distribution and overall trends for the significant associations.

### 3.3 Spectral Profiles of the Wrist Skin Before and After Treatment in Patients with RA

**Fig. 6:**
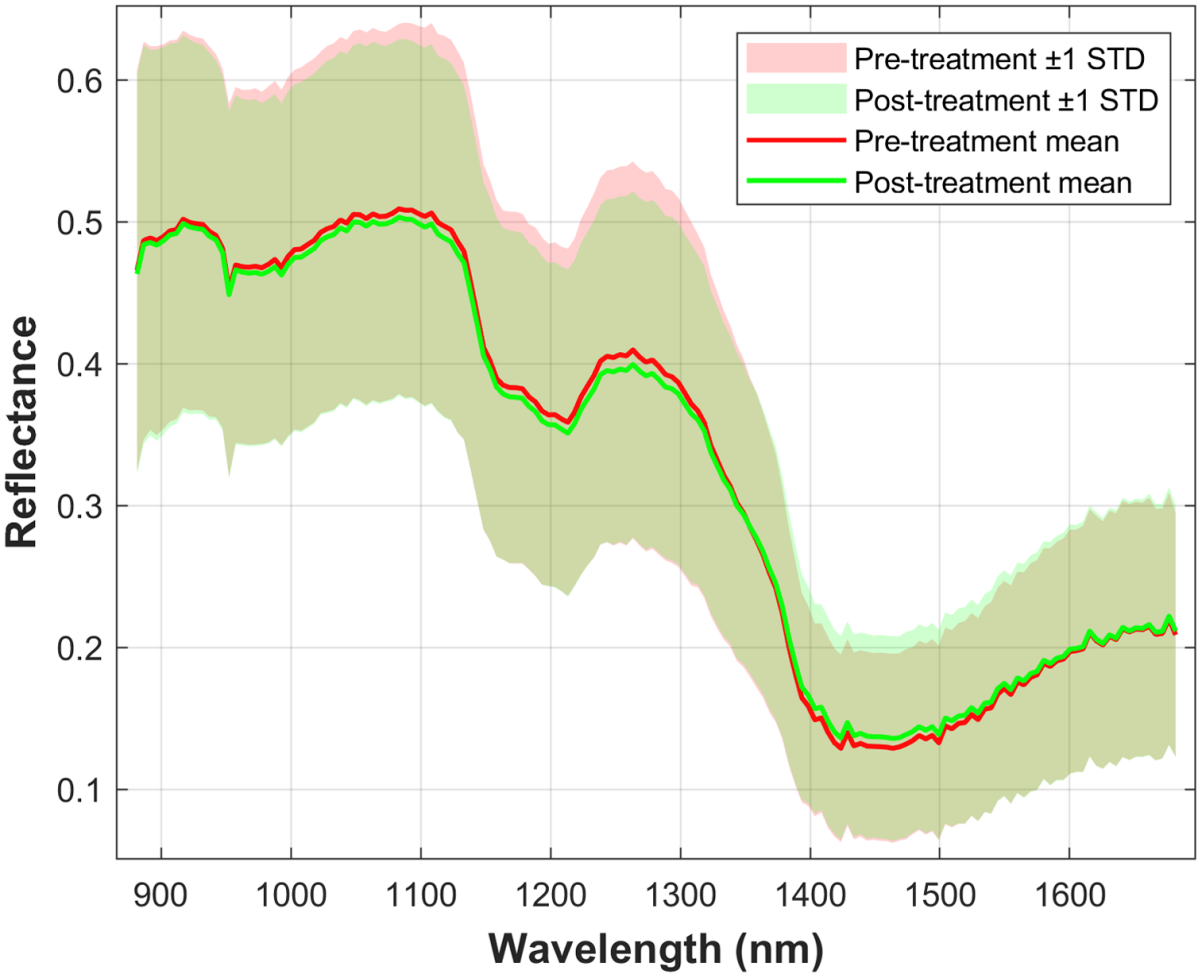
Cohort-level averaged reflectance spectra of wrist skin in patients with rheumatoid arthritis before and after treatment. Solid lines indicate the mean spectra, and shaded regions indicate ±1 standard deviation across the study population.

To provide a direct visualization of treatment-related spectral changes in the short-wave infrared range (900 to 1700 nm), cohort-level averaged reflectance spectra of wrist skin in patients with RA were plotted before and after treatment, as shown in Fig. 6. Shaded ±1 standard deviation bands were included to illustrate spectral variability across the study population.

As shown in Fig. 6, the averaged reflectance spectra exhibited visible pre-/post-treatment differences across the SWIR range, particularly in the regions around 900 to 1100 nm, near 1250 nm, and in the vicinity of 1450 nm. Although spectral differences were observed in all of these regions, the visual difference appeared more pronounced around 1250 nm. The inclusion of variability bands facilitates visual assessment of the pre-/post-treatment spectral differences relative to cohort-level spectral spread. These findings provide a cohort-level visual summary of treatment-related spectral changes in the wrist region.

### 3.4 Intra- and Inter-Subject Hyperspectral Imaging Soft Abundance Score Images and Distribution of Soft Abundance Scores Before and After Treatment in RA Patients

**Fig. 7:**
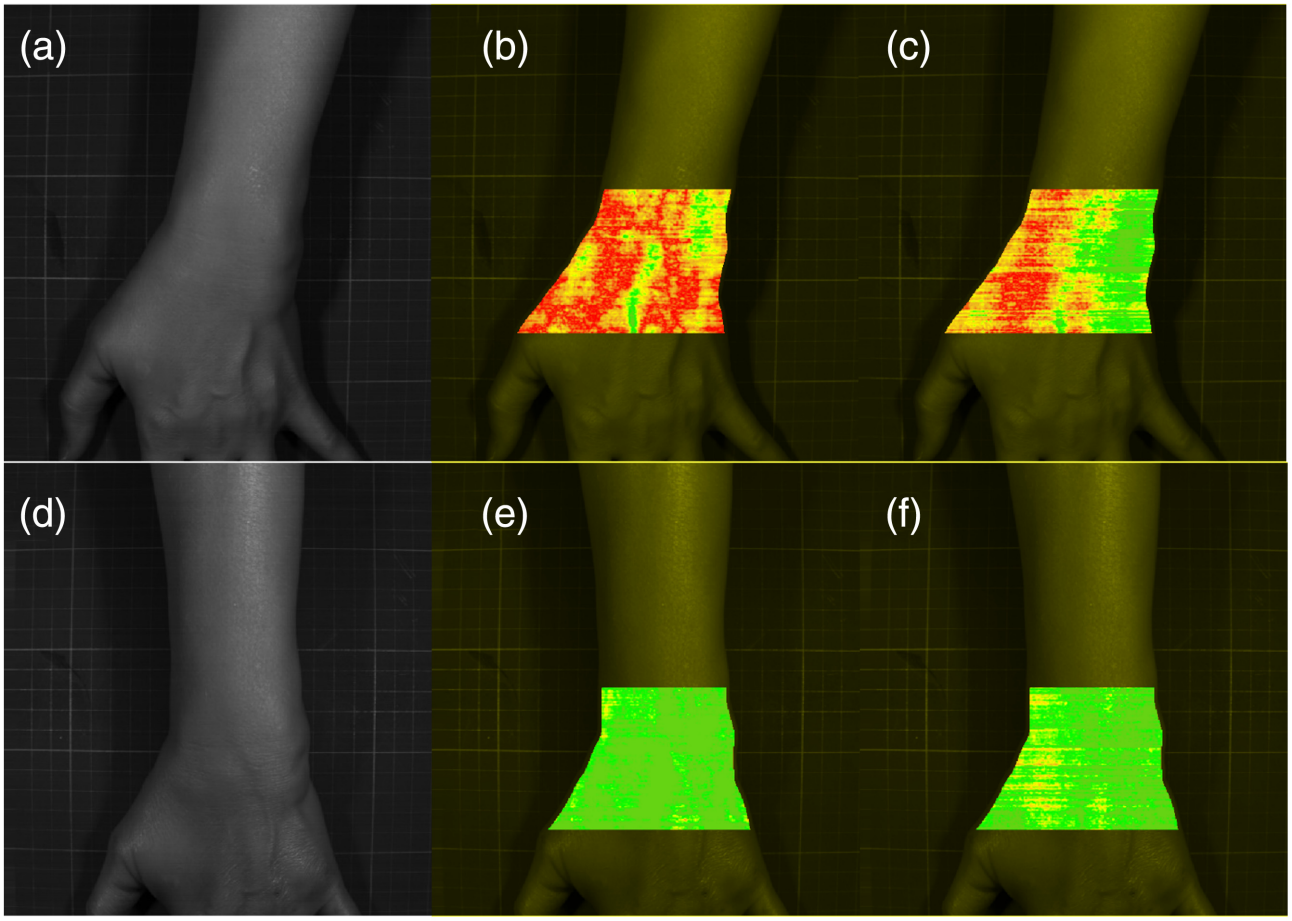
Analysis of the spectral differences before and after treatment in RA patients using intra_HSISAS and inter_HSISAS. (a) Image of the RA patient pre-treatment at the 1303-nm band. (b) Intra_HSISAS abundance score image pre-treatment. (c) Inter_HSISAS abundance score image pre-treatment. (d) Image of the RA patient post-treatment at the 1303-nm band. (e) Intra_HSISAS abundance score image post-treatment. (f) Inter_HSISAS abundance score image post-treatment.

**Fig. 8:**
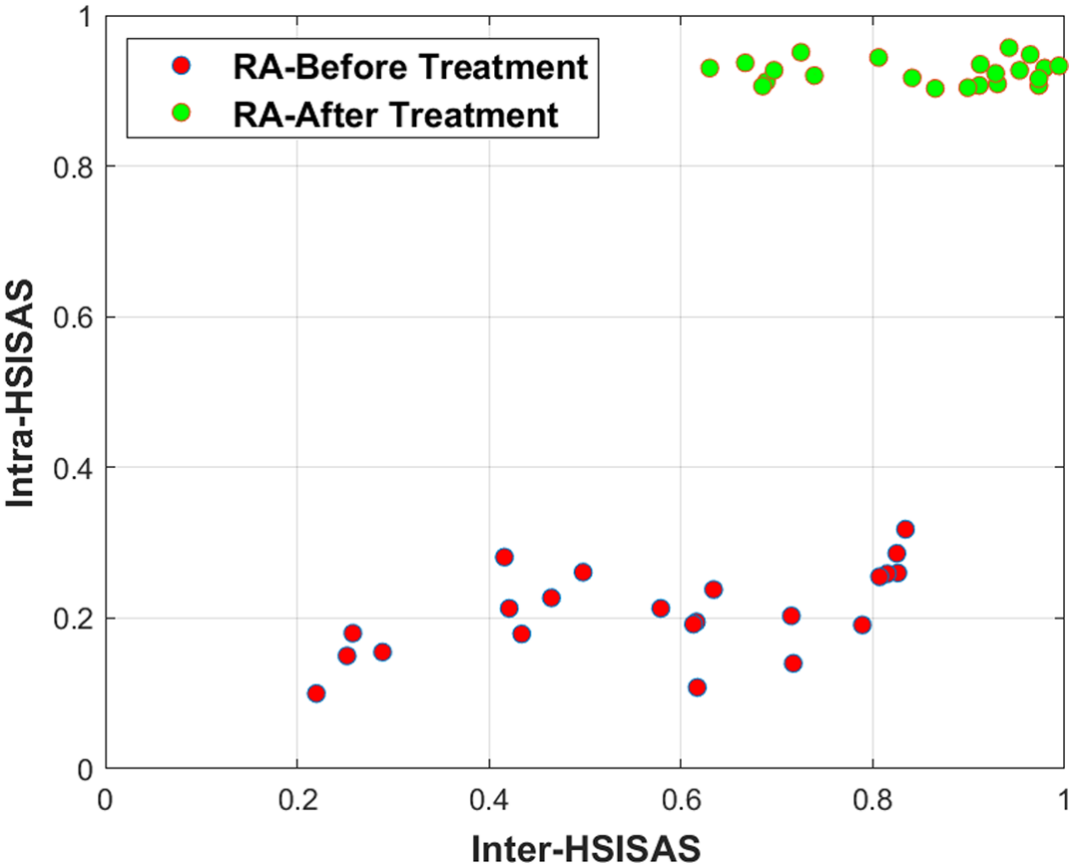
2D projection of the Intra_HSISAS and Inter_HSISAS results for 11 RA patients (22 HSISAS scores for both hands).

Figure 7 shows the results of intra- and inter-subject hyperspectral imaging soft abundance scorer analysis for RA patients before and after treatment. The spectral differences before and after treatment are clearly visualized. In Fig. 7, red represents more severe RA (lower HSISAS values), and green represents milder RA (higher HSISAS values). In addition, Fig. 8 presents a two-dimensional (2D) projection formed by intra_HSISAS and inter_HSISAS, showing clear separation of abundance scores before and after treatment. The pre-treatment clustering is lower, and the post-treatment clustering is higher, indicating that after treatment, the spectral information among RA patients is more consistent.

## 4 Discussion

Previous optical studies^28^^–^^31^ have demonstrated that SWIR spectroscopy (1000 to 2000 nm) enables quantitative assessment of biological tissues, particularly water, lipid, and collagen content. The first overtone of water absorption (1230 to 1380 nm) is especially sensitive to changes in tissue hydration and has been successfully used to monitor skin moisture and to differentiate psoriatic lesions from normal tissue.^28^ This spectral range is also responsive to extracellular water accumulation during inflammation, which results in localized edema and microstructural alterations. Although prior phantom and *ex vivo* studies support the penetration capability of SWIR wavelengths,^16^^,^^17^ the effective sampling depth in diffuse reflectance wrist imaging remains dependent on wavelength, tissue composition, and imaging geometry. Therefore, the present SWIR-HSI measurements should be interpreted primarily as reflecting superficial and subsurface periarticular tissue changes rather than direct signals from deep intra-articular structures.

Accordingly, the observed HSISAS changes should be interpreted conservatively as reflecting a composite optical response from superficial and subsurface periarticular tissues, potentially influenced by soft-tissue edema, local hydration changes, vascular permeability-related changes, and tissue microstructural remodeling associated with RA. In the present study, HSISAS was not designed as an explicit chromophore-fitting model; therefore, the resulting score should not be interpreted as a direct quantitative estimate of water, lipid, or hemoglobin concentration. Rather, it represents a multiband spectral similarity measure derived from the overall reflectance pattern. We therefore consider HSISAS to be useful as a non-contact optical marker of treatment-related tissue change while acknowledging that its physiological specificity remains limited.

In this study, the reflectance spectra of RA patients exhibited prominent variations before and after biologic therapy, particularly near 1250 nm (Fig. 6). These changes are likely related to RA-associated inflammatory processes, including periarticular edema, altered tissue hydration, and microstructural changes. Synovial inflammation increases capillary permeability, which can promote fluid accumulation and tissue swelling.^32^^–^^35^ As a result, tissue optical properties, especially those related to water absorption and scattering behavior, may be altered. The decrease in reflectance near 1250 nm observed after treatment may therefore reflect reduced inflammation-related tissue water content and partial normalization of tissue optical properties, rather than a direct measurement of synovial water content alone. Hence, SWIR hyperspectral imaging appears sensitive to inflammation-associated optical changes in the wrist region.

The improved intra- and inter-subject HSISAS algorithm developed in this study further quantified the treatment-related spectral changes observed in RA wrist tissues. The intra-subject HSISAS (Intra_HSISAS) evaluates treatment-related spectral change within individual patients, whereas the inter-subject HSISAS (Inter_HSISAS) evaluates spectral similarity at the group level. Both indices showed significant increases after treatment, from 0.2 (IQR 0.2 to 0.3) to 0.9 (IQR 0.9 to 0.9) for Intra_HSISAS and from 0.6 (IQR 0.4 to 0.8) to 0.9 (IQR 0.7 to 1.0) for Inter_HSISAS. These increases suggest that the post-treatment spectra became more similar to the reference spectral pattern associated with a relatively improved inflammatory state.

To improve clinical interpretability, correlation analysis was performed using improvement-oriented change variables. Under this sign convention, both Intra_HSISAS improvement and Inter_HSISAS improvement showed positive associations with DAS28 improvement, indicating that greater hyperspectral improvement was associated with greater improvement in clinical disease activity. Inter_HSISAS improvement was also positively associated with PD improvement, indicating that the inter-subject hyperspectral metric showed some concordance with Doppler-related treatment changes. In contrast, ROI_Syn improvement showed a negative correlation with Intra_HSISAS improvement. This finding should be interpreted cautiously, as it may indicate that ultrasound-defined synovial area reduction and HSISAS-based spectral improvement reflect partially overlapping but not identical aspects of treatment response.

The lack of significant correlations between HSISAS improvement and ESR/CRP improvement should also be interpreted cautiously. ESR and CRP are systemic inflammatory markers, whereas SWIR-HSI primarily reflects local wrist-related optical changes in superficial and subsurface periarticular tissues. Therefore, the absence of a significant association may reflect the difference between local optical changes and systemic inflammatory activity, in addition to the limited sample size of this pilot cohort.

The comparison of imaging and laboratory parameters (Table 2) also showed that, except for Doppler energy area, all indices—including synovial area (ROI_Syn), HSISAS scores, DAS28, CRP, and ESR—improved significantly after treatment. The reduction in synovial area from 13,904.8 pixels (IQR 5868.4 to 17,277.5) to 8290.2 pixels (IQR 5646.2 to 17,008.9), together with the decreases in DAS28, CRP, and ESR, generally supports the optical findings and suggests that HSISAS provides complementary quantitative information aligned with established clinical assessments.

In addition, because bilateral wrists were analyzed as separate wrist-level observations, the present analyses may be influenced by within-subject dependence. Therefore, the reported significance levels and correlation estimates should be interpreted with caution, and future studies with larger cohorts should consider patient-level analyses or mixed-effects models to better account for within-subject correlation.

This study represents an exploratory clinical application of SWIR hyperspectral imaging for longitudinal assessment of RA-related tissue changes. The primary advantages of this method include its non-invasive, contact-free acquisition and its high spectral resolution, which together may enable detection of treatment-related optical changes that are not readily captured by conventional imaging modalities. The integration of HSISAS further supports this capability by enabling patient-specific and group-level quantification of treatment-related spectral variability, providing a quantitative optical measure that may be useful for assessing treatment response.

Nevertheless, several limitations should be acknowledged. First, although image acquisition was performed under standardized geometry and calibrated reflectance images were generated for each subject, reflectance SWIR-HSI may still be influenced by surface hydration, specular reflection, and local imaging geometry. Therefore, the present results should be interpreted as reflecting inflammation-related superficial and subsurface periarticular tissue changes, rather than as a direct measure of deep intra-articular pathology or a specific chromophore concentration. In addition, because the target spectrum was defined from post-treatment data within the present cohort, the current HSISAS framework may be susceptible to optimistic bias related to internal self-referencing. Accordingly, the present findings should be interpreted as exploratory rather than as evidence from a fully independent validation framework. Future studies should therefore evaluate independent healthy/reference targets, subject-excluded reference strategies, or external validation cohorts. Second, the small sample size and pilot nature of this study limit statistical generalization and preclude detailed comparison between rheumatoid and degenerative arthritis. Third, only the wrist joints were examined due to the imaging geometry and practical constraints of the HSI platform, whereas ultrasound evaluation required manual annotation by clinicians. Fourth, histological validation (e.g., biopsy) was not performed to directly relate spectral features to tissue composition. Future animal studies and larger patient cohorts could further clarify these relationships. Finally, the HSI system used here is a research prototype with fixed imaging geometry and working distance. Future developments should focus on optimizing optical configurations, improving robustness to acquisition-related variability, identifying key spectral bands, and evaluating whether reduced-band or multispectral implementations can preserve the discriminatory utility of the present approach to support the design of portable, clinically adaptable SWIR-HSI devices.

## 5 Conclusion

This study presents an early clinical application of SWIR hyperspectral imaging combined with intra- and inter-subject HSISAS for longitudinal assessment of treatment-related changes in RA. The proposed approach quantifies both individual- and group-level spectral variations and provides insight into inflammation-associated optical changes in the wrist region. The abundance score maps also offer a practical way to visualize treatment-related spectral changes.

The results indicate that SWIR-HSI with HSISAS may provide a non-invasive and non-contact optical approach for evaluating treatment-related wrist tissue changes in RA. By quantifying reflectance spectral changes associated with inflammation-related tissue alterations, this method may complement existing clinical tools such as ultrasound and laboratory biomarkers. Although further validation in larger cohorts is needed, the present findings support the potential of hyperspectral imaging for future clinical translation in rheumatology, including the development of compact and portable systems for disease monitoring and treatment-response assessment.

## Data Availability

Data supporting the findings of this study are available from the corresponding author upon request.
